# Analyzing the Efficacy and Cost-effectiveness of Anti-platelet Therapy in Unstable Angina/Non-ST Elevation Myocardial Infarction: A Decision Analysis

**DOI:** 10.7759/cureus.5321

**Published:** 2019-08-05

**Authors:** Srikar Reddy, Mevin Mathew, Nimai Patel, Saleh Rahman

**Affiliations:** 1 Internal Medicine, University of Central Florida College of Medicine, Orlando, USA; 2 Medical Education, University of Central Florida College of Medicine, Orlando, USA

**Keywords:** cardiology, decision analysis, decision analysis, non-st elevation myocardial infarction, nstemi, unstable angina, monte carlo, monte carlo simulations, dual platelet therapy, cardiovascular events

## Abstract

Current pretreatment guidelines for coronary angiography in unstable angina (UA) and non-ST elevation myocardial infarction (NSTEMI) involve the use of dual antiplatelet therapy (DAPT: aspirin + adenosine diphosphate (ADP) P2Y12 inhibitor), whereas the use of triple antiplatelet therapy (TAPT: aspirin + ADP P2Y12 inhibitor + GpIIb/IIIa inhibitor) has limited data due to the increased bleeding risk. However, a study directly comparing the efficacy and cost-effectiveness of DAPT vs. TAPT has not been done. A decision analysis was constructed to determine the ideal pretreatment antiplatelet regimen for UA/NSTEMI patients. The parameters were calculated based on published randomized clinical trials. They consisted of probabilities based on a pretreatment strategy (DAPT, TAPT), interventions (percutaneous coronary intervention (PCI), coronary artery bypass grafting (CABG), medical management), and 30-day outcomes (no event, bleeding, vascular event, death). A 10,000 run Monte Carlo simulation provided two outputs: estimated life-years extended and costs for each treatment modality. Quality-adjusted life-years (QALYs) were taken into consideration using calculated coefficients from the literature. The cost/QALY ratio was $1,923/QALY for DAPT vs. $4,734/QALY for TAPT. The use of DAPT for pretreatment was favored (2.46 more cost-effective than TAPT). These results will aid clinicians in providing the most clinically sound and fiscally responsible care for UA/NSTEMI patients.

## Introduction and background

Non-ST elevation myocardial infarctions (NSTEMI) and unstable angina (UA) cause approximately 735,000 people to be hospitalized annually in the United States [1-2]. In such patients, pretreatment strategies for coronary angiography can be challenging for cardiologists. Appropriate pretreatment with platelet aggregation inhibitors may decrease the incidence of ischemic events and thrombosis that would otherwise negatively impact procedures such as percutaneous coronary intervention (PCI) [3]. However, the same pretreatment increases bleeding risk [4]. The challenge for cardiologists is assessing whether the benefit of the decreased ischemia outweighs the bleeding risk for their respective patients. According to the 2014 American Heart Association/American College of Cardiology (AHA/ACC) guidelines for the management of NSTEMI, higher-dose aspirin (162-325 mg) should be used. The guidelines also discuss the potential benefit of adding a P2Y12 inhibitor (e.g. clopidogrel) to aspirin. Guidelines only recommend adding a GpIIb/IIIa inhibitor (e.g. abciximab) if the patient exhibits higher-risk features such as positive troponins [5]. Although many interventional cardiologists are wary of using GpIIb/IIIa inhibitors due to the increased risk of bleeding, there has not been a significant amount of data published in UA/NSTEMI patients corroborating this risk. Two clinical trials, Acute Catheterization and Urgent Intervention Triage Strategy (ACUITY) and Early-ACS, established an increased bleeding risk with GpIIb/IIIa inhibitors without a significant change in 30-day mortality [6-7]. The limitation of these trials is that GpIIb/IIIa inhibitors were used alone and not as an additional treatment to P2Y12 inhibitors. This analysis aims to assess the benefits and risks of the incremental use of GpIIb/IIIa inhibitors as pretreatment for coronary angiography from the cost-effectiveness and clinical standpoints. Such analysis will fill an evidence gap.

## Review

Experimental section

Methods

In order to properly determine the effectiveness of dual antiplatelet therapy (DAPT) vs triple antiplatelet therapy (TAPT) in the management of patients presenting with UA/NSTEMI, a decision analysis model was utilized. For the purposes of this research initiative, DAPT is defined as aspirin + P2Y12 inhibitor and TAPT as aspirin + P2Y12 inhibitor + GpIIb/IIIa inhibitor. In addition, patients in both groups were assumed to have received heparin in accordance with standard interventional cardiology practice.

**Figure 1:**
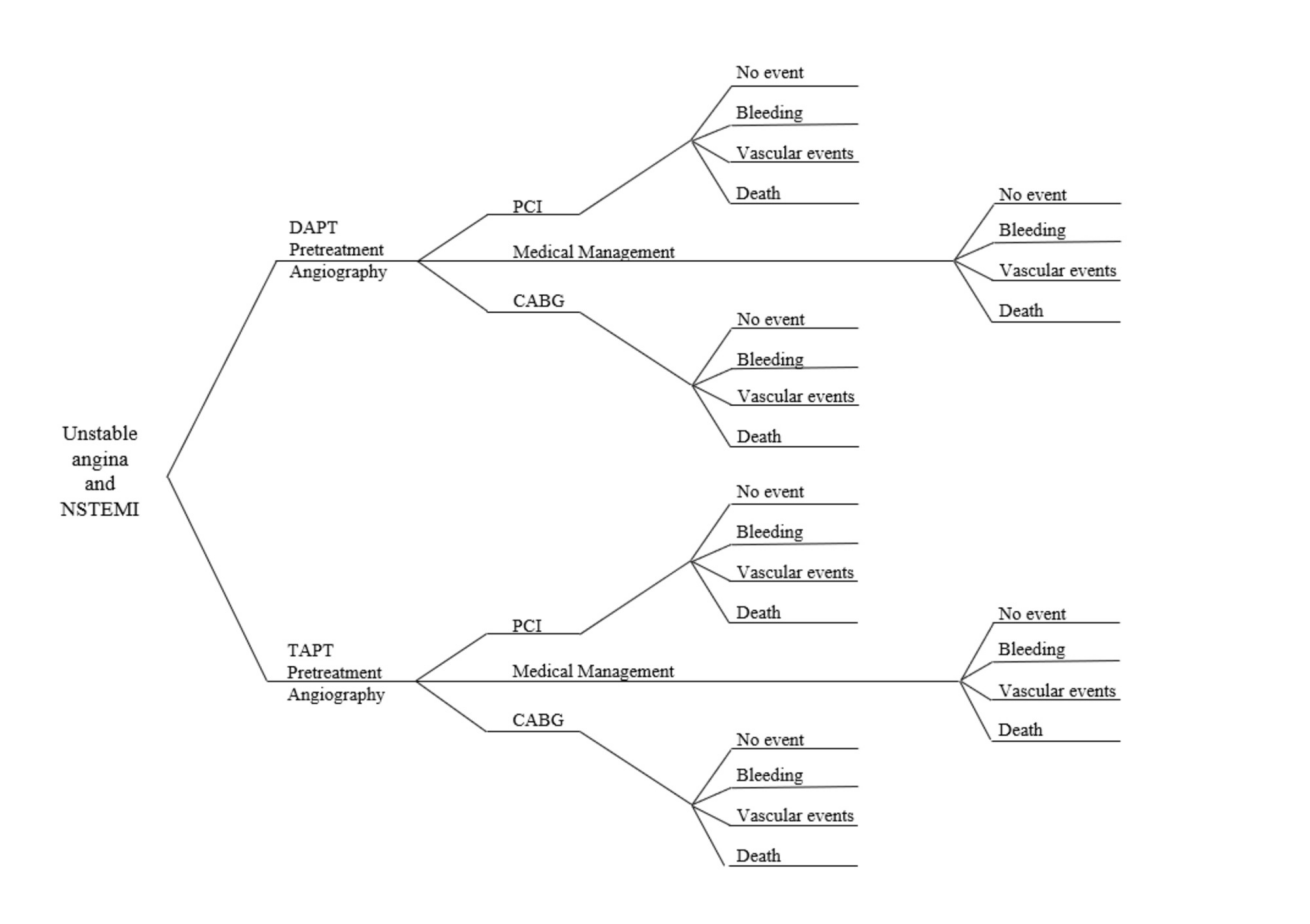
Decision tree with interventions and their possible complications

As depicted in the model below (Figure 1), patients were separated into one of two initial treatment groups based on the combination of antiplatelet regimen received. Thereafter, patients were sorted based on the subsequent intervention in their management. This intervention consists of three different treatment modalities: percutaneous coronary intervention (PCI), coronary artery bypass graft (CABG), or medical management. These treatment options allow for a diverse approach in the analysis of DAPT vs. TAPT in these patients, something that has not yet been extensively studied.

Following our decision analysis model further, it can be seen that each of the four treatment modalities outlined in the previous paragraph had the possibility of leading to one of four clinical outcomes: no significant event, bleeding, vascular pathology, or death (this model assumed that “death” was attributable to any cause and “no event” was considered to be any outcome aside from the other three). Using published randomized clinical trial data, the likelihood of each outcome occurring within 30 days after a specific intervention was calculated. In addition to the estimated probabilities, the predicted costs of each treatment modality, as well as the anticipated complication, were calculated. These costs were based on published literature. With this systematic approach, we were able to conclude the effectiveness of these interventions from the clinical and cost-effectiveness standpoints. The computer program TreeAge Pro 2018 (TreeAge Software, Inc., Massachusetts, US) was used to conduct a Monte Carlo simulation with 10,000 runs of the data.

Model Assumptions

Life-years extended were estimated to be 14.5 years for the no events case, 12.3 years in the bleeding case, and 5.2 years in the vascular-events case [8-10]. Vascular events encompass strokes and re-myocardial infarction. To be clear, life-years extended is different than quality-adjusted life-years. The life-years extended were calculated using a probabilistic analysis after taking clinical trial data into consideration. The probabilities used are mentioned in Table 1 and Table 2 below. The calculation of quality-adjusted life-years involved a separate calculation subsequent to life-years extended. The decision model uses these life-years extended values in accordance with the predicted outcomes (no events, bleeding, vascular events, and death). Therefore, there was no difference in calculating a certain intervention’s life years extended for certain complications. For example, in the bleeding case, the life-years extended value of 12.3 was assumed to be the same for both PCI and medical management. However, the path in reaching the bleeding case can be vastly different because that is contingent on the probability of certain interventions being instituted as well as the probability of such a complication occurring.

**Table 1:** Percent comorbidities associated with DAPT 30 days post UA/NSTEMI PCI: percutaneous coronary intervention; CABG: coronary artery bypass grafting; DAPT: dual antiplatelet therapy; UA: unstable angina; NSTEMI: non-ST elevation myocardial infarction

	CV Events	Bleeding	Death
Medical Management^13^	1.29%	8.59%	3.49%
PCI^12,13,19^	3.61%	3.74%	1.20%
CABG^14-18, 20^	6.93%	6.15%	1.97%

**Table 2:** Percent comorbidities associated with TAPT 30 days post UA/NSTEMI PCI: percutaneous coronary intervention; CABG: coronary artery bypass grafting; TAPT: triple antiplatelet therapy; UA: unstable angina; NSTEMI: non-ST elevation myocardial infarction

	CV Events	Bleeding	Death
Medical Management^13^	1.39%	12.87%	5.38%
PCI^12,13,19^	5.57%	5.96%	1.15%
CABG^14-18, 20^	30.00%	53.00%	5.00%

Any potential vascular events or bleeding that may have transpired between angiography and revascularizations were assumed to be part of the inherent risk associated with a certain management strategy, either PCI, CABG, or medical management. Members of the GpIIb/IIIa inhibitor families were assumed to be of similar efficacy and duration of action. Moreover, it has been established that there is a distinction between the “upstream” use of GpIIb/IIIa inhibitors as opposed to “bail-out” treatment in the event of thrombotic complications during PCI. In this study, the “upstream” case is being assessed.

In this analysis, there was a consideration of both actual life-years extended post-treatment as well as quality-adjusted life-years (QALYs) extended. EQ-5D was the metric used to assess the proper coefficients that should be used within the model to calculate QALYs. The estimates for these coefficients after potential cardiovascular complications, such as myocardial infarctions, were estimated using the Valsartan in acute myocardial infarction (VALIANT) study. In this study, 597 patients had a nonfatal cardiovascular event following an initial MI [11]. These patients were surveyed over a two-year period, and these responses were how the coefficient for QALYs in the event of nonfatal cardiovascular complications was calculated. Similarly, EQ-5D was used to assess the coefficient for bleeding complications following PCI or CABG. Post-discharge bleeding was defined as per the Bleeding Academic Research Consortium (BARC), and EQ-5D was used at the six-month interval. For our purposes in this model, utility scores were assessed whether there was BARC bleeding (BARC 2-4) versus no BARC bleeding or BARC 1; therefore, the utility coefficient applied in the model was binary. The survey that we are referencing focused on BARC bleeding in post-discharge PCI patients [11]. We extrapolated this bleeding utility coefficient to CABG as well and feel this is justified because we are viewing the coefficient in our model as binary with a strict bleeding definition.

Results

Following the Monte Carlo simulation of 10,000 runs, DAPT was shown to be 2.46 times ($1922.6/year vs. $4734.4/year) as cost-effective as TAPT. For DAPT, the predicted life-years extended was 11.5 years, and for TAPT, the predicted life-years was 10.4 years. There was no statistically significant difference between predicted life-years extended by DAPT and TAPT.

Sensitivity Analysis

A two-way sensitivity analysis was performed for each of the following variables: (1) estimated major bleeding risk, (2) estimated mortality risk, (3) estimated vascular events, and (4) estimated risk of having no events.

Pre-treatment Cost-effectiveness

As mentioned earlier, the addition of the GpIIb/IIIa inhibitor did not seem to make a significant clinical difference for patients’ expected life-years extension following medical management, PCI, or CABG. However, the added cost of these drugs makes TAPT less cost-effective as compared to DAPT.

Quality-adjusted Life-years

From the literature search, the QALY post-UA/NSTEMI for no event, bleeding, vascular pathology, and death are 12.04 years, 10.31 years, 3.95 years, and 0 years, respectively [8-10,12-13]. These QALYs were further adjusted in accordance with a certain intervention (CABG, PCI, and medical management); these coefficient adjustments are listed below and were based on literature values (Tables 3-4). 

**Table 3:** Coefficient adjustments for CABG/PCI CABG: coronary artery bypass grafting; PCI: percutaneous coronary intervention

CABG/PCI^12-13^	
No Event	0.86
Bleeding	0.83
Non-fatal CV complication	0.76
Death	0

**Table 4:** Coefficient adjustments for medical management

Medical Management	
No Event	0.45
Bleeding	0.45
Non-fatal CV complication	0.41
Death	0

**Figure 2:**
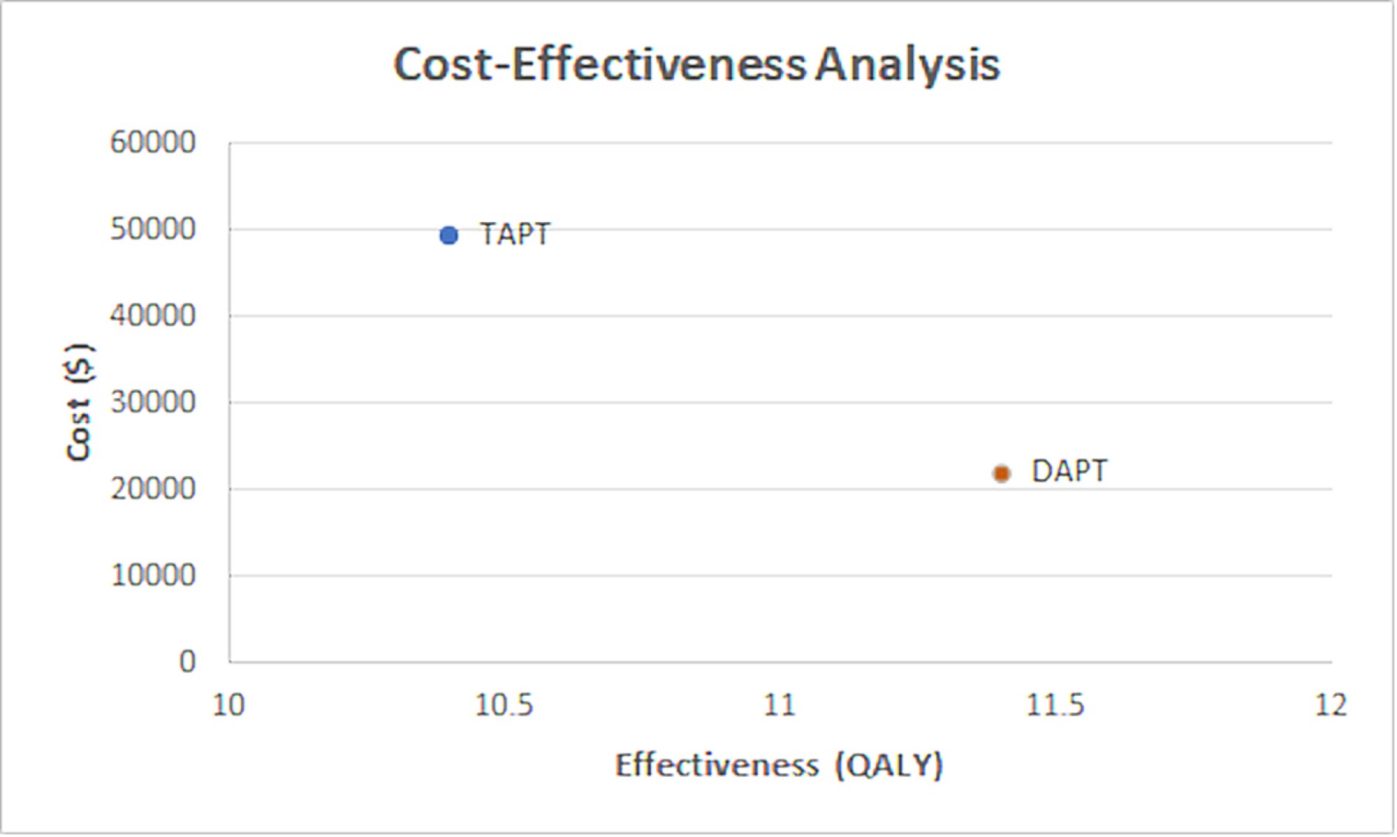
The cost-effectiveness graph comparing the cost and QALYs associated with DAPT and TAPT QALY: quality-adjusted life-year; DAPT: double antiplatelet; TAPT: triple antiplatelet

The cost-effectiveness graph (Figure 2) and comparison of costs table (Table 5) are displayed below:

**Table 5:** Comparison of costs and QALY between DAPT vs TAPT therapy QALY: quality-adjusted life-year; DAPT: double antiplatelet; TAPT: triple antiplatelet

	DAPT	TAPT
Cost	22,022 +/- 18,192	$49,290 +/- 26,588
Effectiveness (QALY)	11.4 +/- 2.1	10.4 +/- 3.1

Discussion 

Based on our decision-analysis model, the use of DAPT is more cost-effective than TAPT in the management of UA/NSTEMI patients. Regardless of the treatment modality, the risk of potential bleeding versus the benefit in controlling life-threatening ischemia must be balanced. If a treatment protocol does not provide sufficient protection against ischemia, there will be a subsequent increased risk of adverse vascular events. With regards to our model, vascular events that manifested in the 30-day period following PCI, CABG, or medical management led to a substantial decrease in QALYs.

Similarly, if a treatment protocol is too potent, there is an increased risk of bleeding. Bleeding in the post-30-day period following initial treatment also leads to a decrease in QALYs, albeit less than in vascular events. However, the risk of bleeding is significantly higher than the risk of vascular events. Therefore, there will be a greater amount of bleeding events that will affect the quality of life for UA/NSTEMI patients.

An important consideration for this decision analysis is the impact on QALYs over just regular life-years extended. Our analysis indicates that DAPT is still superior over TAPT when taking this variable into account. Although the difference in estimated life-years extended between DAPT versus TAPT was not statistically significant, there was a slight decrease in predicted life years for TAPT (10.4) as compared to DAPT (11.4). This is likely attributable to the patient population within the clinical trials. According to the 2014 AHA/ACC guidelines, TAPT is recommended to be used in higher-risk patients; therefore, there is some ascertainment bias. As these patients tend to have a worse prognosis, it follows that their estimated life-years extended would be decreased.

As this model utilized QALY coefficients from survey data, the possibility of intrinsic bias exists. This is because quality of life is inherently a subjective construct. Some bias may have been mitigated due to the large sample sizes of the surveys. Another limitation of this study was the lack of an in-depth analysis of bleeding. Important parameters related to bleeding include the amount, timing, and intervention method. These can all affect patient quality outcomes. There is a lack of published data related to these parameters that could have been referenced for this study.

This decision analysis was done after a compilation of different data sets of varying sizes. There may be some implicit bias because larger studies were assumed to be of equal value to smaller ones. The data gathered was also not geographically limited to the United States (data from various European countries and Australia were included). The use of data sets from other countries may have led to the introduction of additional variables. These countries may have different incidences of UA/NSTEMI and associated complications due to genetic variability and unique environmental influences. Cardiology management strategies may also vary in hospitals in other regions of the world.

Age was solely considered during the prediction of life-years extended; age and race were not used to categorize data. These variables have a great impact on the manifestation and extent of cardiovascular pathology. Once patients in this model were assigned to a specific pretreatment, intervention, and outcome group (ex: DAPT-PCI), there was no shift (ex: DAPT-PCI-no event to DAPT-PCI-bleeding). The use of a Markov model may have addressed this issue.

## Conclusions

While there remains a role for GpIIb/IIIa inhibitors in the management of NSTEMI patients, it is only in situations where the risk posed by ischemia significantly outweighs the bleeding risk. Ideally, there should be an extremely high thrombotic burden. Based on evidence outlined in several clinical trials and AHA/ACC guidelines, they have no role in the management of UA. To conclude, this model and analysis should aid interventional cardiologists in their decision-making process for patients afflicted with UA/NSTEMI.
